# Multilabel Video Classification Model of Navigation Mark's Lights Based on Deep Learning

**DOI:** 10.1155/2021/6794202

**Published:** 2021-11-11

**Authors:** Xu Han, Mingyang Pan, Haipeng Ge, Shaoxi Li, Jingfeng Hu, Lining Zhao, Yu Li

**Affiliations:** ^1^Navigation College, Dalian Maritime University, Dalian 116026, China; ^2^Navigation Mark Department, Changjiang Waterway Bureau, Wuhan 430010, China

## Abstract

At night, buoys and other navigation marks disappear to be replaced by fixed or flashing lights. Navigation marks are seen as a set of lights in various colors rather than their familiar outline. Deciphering that the meaning of the lights is a burden to navigators, it is also a new challenging research direction of intelligent sensing of navigation environment. The study studied initiatively the intelligent recognition of lights on navigation marks at night based on multilabel video classification methods. To capture effectively the characteristics of navigation mark's lights, including both color and flashing phase, three different multilabel classification models based on binary relevance, label power set, and adapted algorithm were investigated and compared. According to the experiment's results performed on a data set with 8000 minutes video, the model based on binary relevance, named NMLNet, has highest accuracy about 99.23% to classify 9 types of navigation mark's lights. It also has the fastest computation speed with least network parameters. In the NMLNet, there are two branches for the classifications of color and flashing, respectively, and for the flashing classification, an improved MobileNet-v2 was used to capture the brightness characteristic of lights in each video frame, and an LSTM is used to capture the temporal dynamics of lights. Aiming to run on mobile devices on vessel, the MobileNet-v2 was used as backbone, and with the improvement of spatial attention mechanism, it achieved the accuracy near Resnet-50 while keeping its high speed.

## 1. Introduction

In recent years, various AI technologies have been utilized to research smart ship [1, 2] and intelligent navigation [3, 4], among which intelligent sensing of navigational environment is the first important ability [5]. For collecting information of navigational environment, addition to the traditional ECDIS (Electronic Chart Display and Information System), Radar, and AIS, recognizing navigational environment from camera became a new way and challenge. It is expected to supplement effectively the autonomous sensing abilities of vessel, even finally replace the bridge watchkeeping.

For navigation safety, the most important objects should be recognized include the dynamic ship objects and navigational features, such as channel and restricted areas. Generally, these features are marked by aids to navigation, which consist primarily of buoys and beacons [6]. Both floating buoys and fixed beacons may be collectively called “navigation marks”; they have distinctive shapes, colors, top marks, and other auxiliary markings, which can be observed during daytime to indicate their purpose. Navigation marks also have different lights to be identified at night.

For intelligent sensing of navigational environment, many studies about ship classification and detection based on deep learning techniques have been carried out gradually [7, 8]; however, few research focused on the recognition of navigational features so far. In 2019, we started a research on the recognition of navigation marks during daytime and proposed a classification model based on ResNet-50 and a multiple scale attention mechanism [9]. In this article, we research the recognition of navigation marks at night features.

During daytime, the recognition of navigation marks can be performed by observing their shape, color, and other visual features. However, at night, navigation marks disappear, and the methods by observing their appearance from image will be not working anymore. In this case, the lights on navigation marks become the most important feature to identify and ascertain their purpose.

Different with road traffic light, the lights on navigation marks are distinctive in both color and flashing characteristic. For example, the light of port lateral marks may show two red flashes within a 5 seconds period, and the light of starboard lateral marks may show two green flashes within a 5 seconds period. So, to identify different type of the lights, video instead of single image is needed. Furthermore, there are many types of lights, which are defined by the combination of four types of color (red, green, yellow, and white) and more than ten types flashing characteristics. Obviously, the classification of navigation mark's lights can be regarded as multiple labels classification problem.

Therefore, this article studied the visual recognition of navigation mark's lights based on video and multilabel classification methods, and its contributions are summarized as follows:

1. Utilizing deep learning technologies to recognize the lights on navigation marks at night.
2. A novel method is proposed to extract the flash characteristic of navigation mark's light. We use the V channel of the image as the input to complete the recognition of flash characteristic.
3. We design a feature extraction network for the color characteristic of navigation mark's light based on the attention mechanism. Meanwhile, we use the lightweight model as the baseline model. The new model has better results in speed and accuracy.
4. Finally, we propose an NMLNet (Navigation Mark's Light Network) to capture the light features including color and flashing characteristic from video for the classification of navigation mark's lights.

The remainder of the article is organized as follows.  Section 2 provides the overview of related researches.  Section 3 presents the proposed models for the classification of navigation mark's lights. In Section 4, the experimental results are analyzed and discussed. The conclusions and future works are given in Sections 5 and 6.

## 2. Related Works

### 2.1. Recognition of Traffic Light

In some extent, the lights on navigation marks have similar color characteristic with traffic lights. The accurate recognition of traffic lights is also an important part of both driving assistance systems and autonomous vehicles.

There are two types of methods for traffic light recognition: model based and learning based [10]. As traffic lights have a well-defined structure in terms of color and shape information, most earlier methods for traffic light detection and recognition were model based [11]. For example, Diaz et al. (2012) presented a technique to detect suspended traffic lights based on colors and features, such as black area of traffic lights or area of lighting lamps [12]. Zhang et al. (2014) proposed a model for traffic light recognition, which was built by the combination of color, shape, and geographic information [13]. However, because the model-based methods were not robust when assumptions were not strictly observed, learning-based methods especially deep learning methods were introduced. Fernandes et al. (2014) employed CNN (Convolutional Neural Network) to recognize the state of traffic light [14]. Weber et al. (2014) proposed a camera-based system for real-time detection and classification of traffic lights based on CNN, called DeepTLR [15]. Jensen et al. (2017) applied the YOLO (You Only Look Once) to detect traffic lights based on the public LISA Traffic Light data set [16]. Behrendt et al. (2017) studied a deep learning approach to traffic lights and proposed a traffic light detector and a traffic light tracker [17]. Muller and Dietmayer (2018) presented a deep learning approach based on SSD (single shot detection) for traffic light detection and achieved high accuracy on the DriveU Traffic Light Dataset [18]. Compared with the model-based methods, the learning-based methods tend to be more robust, especially, as the large annotated data set of traffic lights are becoming publicly available and the performance of deep learning-based methods is improved greatly over previous methods.

According to the experiment on the recognition of traffic lights, deep learning-based method is selected to research the recognition of navigation mark's lights in this article. However, due to the navigation mark's lights have both color and flashing characteristic, more complex model with regard to video and multilabel classification is needed.

### 2.2. Video Classification

For video classification, there is a recent trend to learn feature representations with deep learning from video data. A commonly used method is to treat a video clip as a collection of frames, features of each frame are captured by a deep CNN model, such as CoogleNet, VGGNet, and ResNet, and then averaged into representations of the video, which are finally inputted to standard classifiers, such as SVMs for recognition [19]. There are also many works focusing on applying end-to-end CNN models to learn hidden spatiotemporal patterns for video classification [20]. A. Karpathy et al. (2014) provided an extensive empirical evaluation of CNNs on large-scale video classification and explored CNNs that account for temporal connectivity in videos [21]. Tran et al. (2015) also proposed a simple approach for spatiotemporal feature learning using deep 3D convolution [22].

However, because 3D CNN and spatiotemporal patterns in video are too complex, the training of the end-to-end is usually time consuming. Simonyan and Zisserman (2014) proposed a two-stream approach, which breaks down the representation learning of video into two separate parts, a spatial CNN for spatial feature learning and a temporal CNN for temporal clues [23]. Wang et al. (2016) presented a framework for action recognition in videos and proposed a temporal segment network-based ConvNet, which operates on a sequence of short snippets sparsely sampled from the entire video instead of working on single frame, to tackle the inability of the traditional two-stream ConvNets in modeling long-range temporal structure [24]. Carreira et al. (2017) introduced a new two-stream inflated 3D ConvNet, I3D, in which filters and pooling kernels are expanded into 3D to learn spatiotemporal features from video [25]. However, it is hard for ConvNet to tackle complicated actions happening over a long time due to failing to consider the order of frames [20]. Therefore, recently, RNN (Recurrent Neural Network) is used in many researches to account for the temporal dynamics in videos. Wu et al. (2015) presented that LSTMs (Long Short Term Memory) and CNNs are highly complementary and fused them to jointly model spatiotemporal clues for video classification [26]. Donahue et al. (2017) proposed a Long-term Recurrent Convolutional Networks for video recognition problems, in which a CNN processes the variable-length visual input and its outputs are then fed into an LSTM to produce a variable-length prediction finally [27].

The attention mechanism has been leveraged generally in deep learning because it enables a neural network to focus on a subset of inputs that are directly related to the targeted semantic class. For video classification, the attention mechanism is also helpful to identify the most discriminative spatiotemporal volumes. Sharma et al. (2015) proposed an LSTM-based action recognition model with a soft-attention mechanism to highlight the learned relevant parts in video frames [28]. Li et al. (2016) applied motion-based attention derived from optical flow images in convolutional LSTM models to discover relevant spatiotemporal volumes and achieve better action localization [29].

According to the research trend on video classification, to classify navigation mark's lights accurately and efficiently, two-stream framework with long-range temporal structure and attention mechanism was selected.

### 2.3. Multilabel Classification

Multilabel classification means the task has more than two target labels, and its methods can be grouped into two strategies: problem transformation and adapted algorithm. Problem transformation tries to transform multilabel problem into single label problem by the way of binary relevance or label power set, and adapted algorithm extends and adapts existing specific learning algorithms to handle directly the multilabel problem [30], such as multilabel *k* nearest neighbors (ML-KNN) and multilabel decision tree (ML-DT) [31].

Deep neural network can be used as powerful classifier in the problem transformation methods, and it is also powerful enough to be adapted directly to tackle multilabel classification problems. Jesse Read and Fernando Perez-Cruz (2014) used a deep learning approach with Restricted Boltzmann Machines for a variety of multilabel classification contexts, which outperformed a number of competitive methods in an empirical evaluation [32]. Maxwell et al. (2017) presented a multilabel classification method based on deep learning classifier to predict chronic diseases, such as diabetes, hypertension, and fatty liver, in patients, and the results showed that it gave much higher accuracy than SVM and ML-KNN [33]. Baltruschat et al. (2018) cast the pathology detection into a chest X-ray multilabel classification problem and investigated several CNN-based approaches by adapting the last dense layer to match the label number and adding a sigmoid activation function; the result showed that an optimized architecture based on ResNet achieved best performance [34]. Janwe et al. (2018) presented an approach for automatic, multilabel, semantic, video concept detection, using an adapted deep CNN and a foreground-driven concept co-occurrence matrix (FDCCM) based on TRECVID video data set [35]. Jabreel et al. (2019) presented a deep learning-based system for multiple emotion classification in Twitter, in which deep learning was exploited to solve the transformed binary classification problems [36]. Liu et at. (2019) proposed an LSTM-based network structure to detect multilabel events in a given surveillance video data set, and the experimental results showed that it outperform the SVM-based method for visual event detection [37].

In the context of classification of the lights on navigation marks, their features can be mapped into two labels, color and flashing. Obviously, deep neural network would be the best classifier; however, which strategy to adopt deep neural network is still needed to investigate and compare further. The binary relevance approach has disadvantage of training multiple classifiers, but in this case, there is only two labels without obvious correlation. The label power set approach is hard to scale for large number of label combinations, but it works fine with lesser combinations. The adaptive method may be the most powerful modeling ability, but it is also harder to train. So, in this article, three different models based on binary relevance, label power set, and adapted algorithm were investigated and compared to find the best method to tackle the multilabel classification problem of navigation mark's lights.

## 3. Classification of Navigation Mark's Lights

Before introducing the multilabel video classification model of navigation mark's lights, the classification standard of navigation mark's lights is described.

### 3.1. Labels for Classification of Navigation Mark's Lights

There are three features to describe the lights on navigation marks: color, phase, and period. Colors of lights include white (*W*), red (*R*), green (*G*), and yellow (*Y*). Phase is the light changing pattern within a compete cycle. Period is the time in seconds of a complete cycle. These features of light are noted on the nautical charts next to the light in a form such as “FI *R* (2) 5s.” Here, “FI” is a type of phase characteristic, “*R*” is the red color of light, and “(2) 5s” means the light flashes twice every 5 seconds.  Table 1 lists the most common types of light's phrase characteristic.

Navigation mark has different light to ascertain its purpose. Under IALA A, the port lateral mark may have a light of “FI *R* (2) 5s,” and the starboard lateral mark may have a light of “FI *G* (2) 5s”. There are four cardinal marks if lighted use white quick flash lights: the north cardinal mark has continuous quick flash “*Q*”, the east cardinal mark has a light of “*Q* (3) 10s,” the south cardinal mark has a light of “*Q* (6) + LFI 15s,” and the west cardinal mark has a light of “*Q* (9) 10s”; they can be easily remembered from thinking of a clock dial. For the marks indicating isolated dangers, the light consists of a white flash such as “Fl (2) 5s”. Safe water marks typically have a white flashing light with Morse code A, “Mo (*A*) W 10s”. For the special purpose marks, they may have yellow lights with any phrase characteristics that is not used for white lights, for example, “FI (4) Y 8s”. In China, there are seven types of special marks for different purposes, such as anchorage, prohibited area, and so on, they are typically lighted with different Morse code, such as *Q* (“Mo (*Q*) Y 12s” for anchorage), P (“Mo (P) Y 12s” for prohibited area), and so on.

The recognition of lights is most important way to identify different type of navigation marks at night. In this article, the classification of navigation mark's lights was treated as multilabel classification problem. The color label has four values of red (R), green (G), yellow (Y), and white (W). The phrase characteristic and period were combined as flashing label, for example, “FI (2) 5s” should be labeled as a flashing time series “0.5s (flash) + 1.0s (dark) + 0.5s (flash) + 3.0 (dark)” in practice.  Table 2 shows the flashing time series of some different flashing labels.

### 3.2. Model Based on Binary Relevance

#### 3.2.1. Network Structure

**Figure 1:**
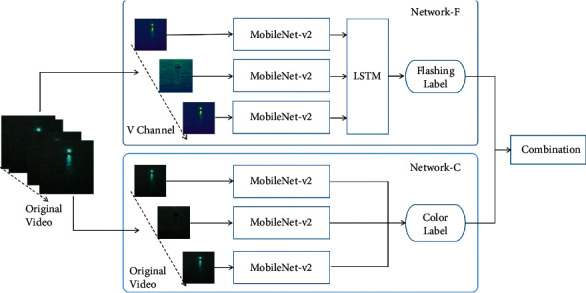
Network structure of model-based binary relevance.

In the network of model-based binary relevance, there are two branches for the classifications of color and flashing, respectively. As shown in Figure 1, Network-C is the classifier for color label, and the Network-F is the classifier for flashing label. The two labels are finally comb to obtain the category of navigation mark's light. The calculation formula is shown in equation (1), and the Network Structure flow is shown in Algorithm 1.


(1) $$\begin{array}{c} Y=F\left(\text{LSTM}\left(X^{V}\right)+X^{\text{RGB}}\right),\\ X_{i}^{V}=\text{MobileNet}-V2\left(X_{i}^{V}\right),\\ X_{i}^{\text{RGB}}=\text{MobileNet}-V2\left(X_{i}^{\text{RGB}}\right). \end{array}$$

*Y* is the label of navigation mark's light, *X*^*V*orRGB^ is the V-Channel or RGB format Original Video, *X*_*i*_^*V*orRGB^ is a frame in *X*^*V*orRGB^, and *F* is merge labels.

In the Network-C, a deep convolutional neural network (MobileNet-V2) is used to extract the color features from very frame of the original video. For the case of navigation mark's lights, colors are divided into five categories: green, red, white, yellow, and none. So, the color label of a video segment will be marked as a sequence of color, such as [red, red, red, none, none, none, red...]. And, before the final label combination, the color sequence will be further summarized as a single label that is the most frequent color except “none”.

Network-F has a two-part architecture, the spatial part MobileNet-V2 is used to capture the brightness characteristic of lights in each video frame, and in the temporal part, LSTM is used to capture the temporal dynamics and output the flashing label such as “FI(1) 4s.”

**Figure 2:**
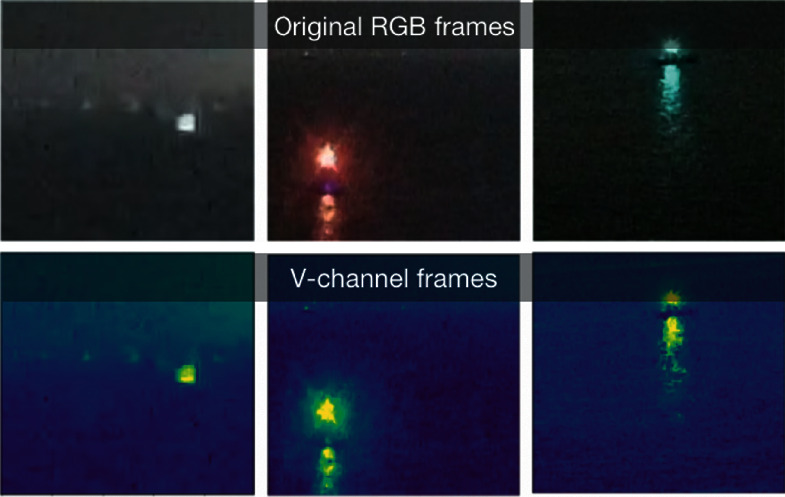
V-channel of video frames.

For MobileNet-V2, the original video input is replaced by its V-channel portion. Every frame of the original video is a color image with red (R), green (*G*), and blue (B) channels; however, to extract the light flashing characteristics, the RGB color is too redundant, the HSV color model with hue (H), saturation (S), and value (V) channels is more suitable to eliminate redundant information. In HSV color space, “H” is the color portion, “*S*” presents the amount of gay in a particular color, and “*V*” describes the brightness of the color. Just the brightness information of V-channel is enough to extract the flashing characteristics of navigation mark's light. So, to reduce the network's parameters, each frame of the original video is converted to HSV model, and only V-channel is retrained as Figure 2 shows.

#### 3.2.2. Spatial Attention

In order to be able to be applied in mobile device on vessel, MobileNet-v2 with high computation speed is used as the backbone of both Network-C and Network-F for feature backbone of both Network-C and Network-F for feature extraction. However, the accuracy of MobileNet-v2 is lower than ResNet and other more complex network structures. So, it is necessary to improve classification accuracy using some tricks such as attention mechanism, which is proved helpful to identify the most discriminative subset of inputs.

The attentional mechanism in the image classification in order to make the model focus on the relevant information and ignore irrelevant information. Spatial attention and channel attention are two important technologies. Spatial attention is to find the regions of the feature map that are most relevant to the task. Channel attention is assigning weight to different channels. For this study, it is crucial to find the location of the navigation mark's light. Therefore, we optimized MobileNet-v2 by the spatial attention.

**Figure 3:**
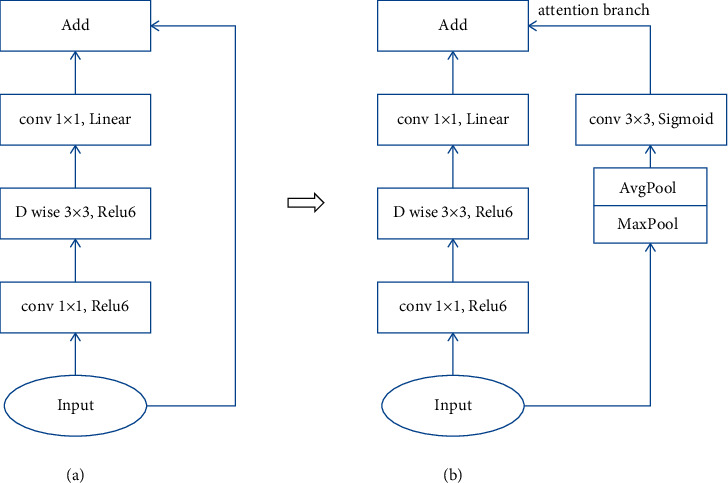
Improvement of Inverted residual module with attention branch. (a) Original module. (b) Improved module.

**Figure 4:**
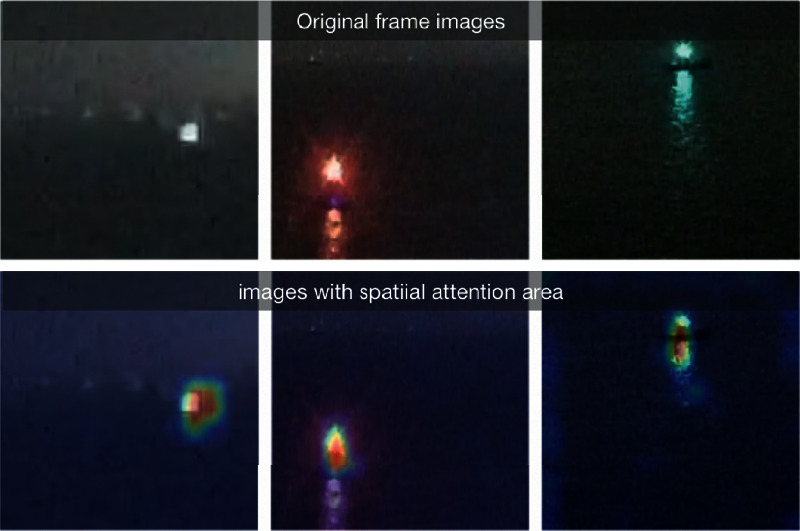
Effect of the spatial attention mechanism.

Figure 3(a) shows the original inverted residual module of MobileNet-v2, and Figure 3(b) shows the improved module in which a spatial attention branch is added. The new attention branch includes a maxPool layer, avgPool layer, and a conv 3 × 3 layer, which has Sigmoid as activation function. It would give each feature a corresponding weight and let the final feature vector map more effectively on the target areas.  Figure 4 shows the effect of spatial attention mechanism, the above are original images, and the bottom ones describe the attention areas captured by the improved MobileNet-v2. From the attention area pictures, it can be observed that the lights and their water reflections are both focused on.

The calculation formula is shown in equation (2):

(2) $$\begin{array}{c} y_{1}=F_{1}\left(x\right)+x,\\ y_{2}=F_{1}\left(x\right)+F_{2}\left(x\right), \end{array}$$

where *y*_1_is original module, *y*_2_ is improved module, *F*_1_ is conv1 × 1(Relu6) + *D* wise3 × 3(Relu6) + conv1 × 1(Linear), and *F*_2_ is MaxPool + AvgPool + *D* wise3 × 3(Sigmoid).

### 3.3. Model Based on Label Power Set

**Figure 5:**
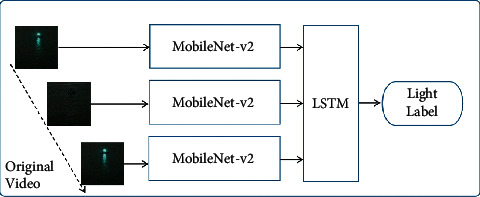
Model based on label power set.

In this model, the color and flashing label are combined into a unique light label associated to one class such as “Fl *R* (2) 10s,” and only one network is needed to train all unique light labels. As Figure 5 shows, the network also has a two parts structure of MobileNet-v2 + LSTM. The MobileNet-v2 has 3-channel RGB images as input, and it is also improved with spatial attention mechanism. The LSTM has an input of a sequence of color output from all MobileNet-v2 parts, and a final light label probability vector is output through a softmax layer.

### 3.4. Model Based on Adaptive Algorithm

This model has the same network structure as the one based on label power set. However, the output vector of softmax layer is not about all combination labels but the concat of color label and flashing label.

**Figure 6:**
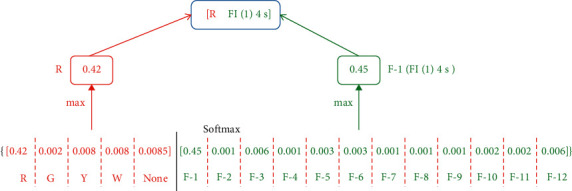
Softmax layer of the model based on adaptive algorithm.

Figure 6 shows the diagram of the softmax layer. Color labels and flashing labels (see Table 2) are concated to form an array, and the sum of probability generated by softmax for each elements is 1. The maximum probabilities of color and flashing parts in the array are leveraged, respectively, to pick up the final color label and flashing label.

## 4. Experiments and Results

### 4.1. Data Set of Navigation Mark's Lights

#### 4.1.1. Data Set Information

**Figure 7:**
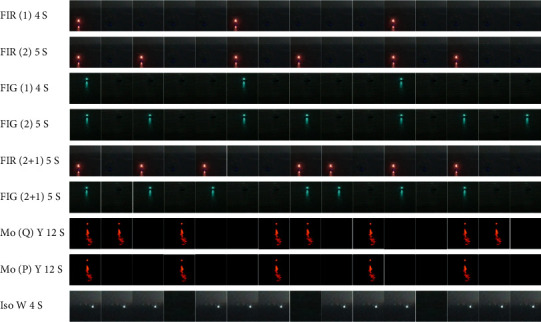
Data set of navigation mark's lights.

8000 minutes video of 9 types of navigation mark's light have been collected. The 9 lights, FI R(1) 4s, FI G(1) 4s, FI R(2) 5s, FI G(2) 5s, FI R(2 + 1) 6s, FI G(2 + 1), Mo(Q) Y 12s, Mo(P) Y 12s, and Iso W 4s are shown in Figure 7. The videos of each type are then divided into training and validation data set, with a ratio of 8 : 2, about 7200 minute video was used for model training, and about 1800 minute videos was used to verify accuracy.

#### 4.1.2. Video Cutting and Slice

As the lights are periodic in the videos, in order to get uniform input format for the classification models, all video samples are cut into segments with 12s. 12s is the longest period of lights in the data set. So, every segment includes at least one whole period of a light but may not always from its initial phase.

Furthermore, in order to speed up training and increase generalization capability, the models are not trained with every frame of the video segments. As the shortest flashing period is 0.4s in the data set and the videos have a frame rate of 25, every video segment is sliced every 8 (0.4×25) frames and formed a 38 images sequence as input. Also, every image has a size of 224 × 224 layer.

### 4.2. Experimental Design

All the proposed models are implemented by Python3.7 and the deep learning framework of Pytorch1.7.1 and trained in a workstation with one graphics card of NVIDIA GeForce GTX 2070 SUPER.

In the training process, all models use the same optimizer of SGD and the same loss function of CrossEntroy and utilize the Pytorch's pretrained weights on Imagenet, which was verified and can speed up the convergence in practice.

For the experiment, we use four evaluation indexes to access the model: Acc, Params, Latency_time (CPU), and Latency_time (GPU). Acc represents the accuracy of the model, Params represents the number of parameters of the model, Latency_time (CPU) represents the running speed of the model in the CPU environment, and the Latency_time (GPU) is in the GPU environment.

### 4.3. Experimental Processing

**Figure 8:**
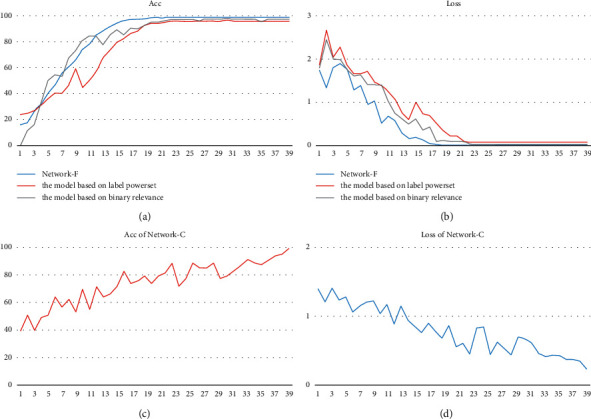
Training curves: (a, b) the Acc and Loss curves of Network-F, the model based on label power set and the model based on adaptive algorithm. (c, d) The Acc and Loss curve, respectively, of Network-C.

Figure 8 shows the training curves, respectively, of the model based on binary relevance, the model based on label power set and the model based on adaptive algorithm. In the model based on binary relevance, Network-C and Network-F were trained independently.

From the training curves, it can be found that the convergence speed of the Network-C is slower that other models with LSTM part. As a special recurrent neural network model, LSTM can learn effectively the semantic relationship between frames. However, in Network-C, without LSTM, there is not semantic related feature to help its classification.

### 4.4. Results

The test results on the validation set are compared in Table 3.

It can be found that the model based on binary relevance is better than others in all evaluation indicators, including accuracy (Acc), parameter numbers (params), and computation speed (latency time). Also, in order to investigate the effect of spatial attention mechanism, with the model structure that based on binary relevance, the performance of three different backbone networks was compared. The experiment results are shown in Table 4.

The following can be observed from the comparison: (1) due to its compact network structure, MobileNet-v2 has lower accuracy than ResNet-50, but it has less parameters and higher computation speed, and (2) with spatial attention mechanism, the improved MobilesNet-v2 achieves the same level accuracy as Resnet-50 while keeping its high efficiency.

Obviously, from the comprehensively consideration of accuracy and speed, the multilabel model based on binary relevance and improved MobilesNet-v2 with spatial attention mechanism has the best performance. Also, it was selected as the final model called NMLNet to classify the navigation mark's lights.

### 4.5. Analysis

The binary relevance approach has obvious disadvantages in many cases. But for the classification of navigation mark's lights, the two labels about navigation mark's light have no obvious correlation. Instead of increasing the complexity and reducing accuracy, the separate training of color and flashing label brings several merits.

1. It reduces complexity of the network for capturing flashing characteristic by simplifying input from three RGB channels to one V-channel, which is also helpful to improve accuracy of flashing classification.
2. It increases accuracy of color classification by training samples from all types of lights of which result can be combined with any flashing phase.
3. The label combination mechanism makes that it has higher generalization ability, which means even if there is no any data sample of a new light, but when the light has same flashing with an existing light and same color with another light, it still can be recognized correctly. This merit is very practical especially when there are not sufficient samples of all types of lights.

**Figure 9:**
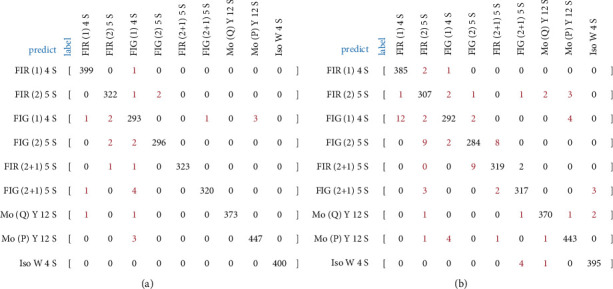
Confusion matrix of misclassified lights. (a) NMLNet. (b) Model based on label power set.

Figure 9 shows the confusion matrix on the validation set, of NMLNet and the model based on label power set, respectively. It can be found that, in NMLNet, there is few color misclassification; however, in the model based on power set, there are much more color misclassifications such as from FI R(1) 4S to FI G(1) 4S and from FI R(2 + 1) to FI G(2 + 1).

**Figure 10:**
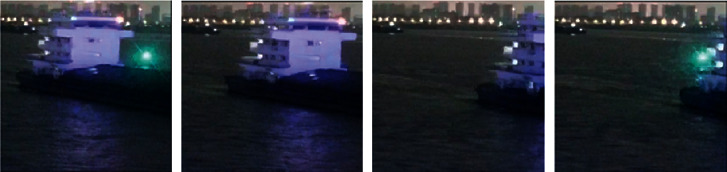
Light is blocked by moving vessel.

In NMLNet, there are several misclassifications of flashing phase for example from FI G(1) 4s to FI G(2) 5s or from FI G(1) 4s to FI G(2 + 1) 5s. By investigating into the video segments in detail, it was found that the light was blocked by moving vessel in some frames as Figure 10 shown. However, this scenario was not considered in NMLnet, in order to further promote the classification accuracy, how to treat this kind of occasional event efficiently should be studied in the future works.

Aiming to run on mobile devices or computers with weak computing capabilities on vessel, instead of ordinary classification models such as Resnet-50, a lightweight network with smaller parameters, MolibleNet-v2, was selected as the backbone of NMLNet. Although MolibeNet-V2 has lower accuracy than Resnet-50, the improved MobilesNet-v2 with spatial attention mechanism can achieve the accuracy near Resnet-50 while keeping its high recognition speed. Lightweight will continue to be the main direction of optimization.

## 5. Conclusion

At night, the familiar scene of buoys and other navigation marks disappear to be replaced by fixed and flashing lights. Navigation marks are seen as a set of lights in various colors rather than their familiar outline. Instead of natural visual interpretation that can be seen in daylight, marks are code at night. So, navigation at night means that navigator must decipher the meaning of the lights. Translating the code of the lights into meaningful information adds to the navigator's workload considerably, and especially, it is a big challenge for the less experienced.

This article studied the intelligent recognition of lights on navigation marks at night based on deep learning methods. As the characteristics of navigation mark's lights include both color and flashing, the recognition problem was treated as a multilabel classification problem based on video. Three different multilabel classification models, based on binary relevance, label power set, and adapted algorithm, respectively, are investigated and compared.

Experiment was performed on a data set with 8000 minutes video, and the results show that the model based on binary relevance has the highest accuracy about 99.23% to classify 9 types of navigation mark's lights, it also has the fastest computation speed with least network parameters. So, for navigation mark's lights, the model based on binary relevance called NMLNet is the best choice especially when there are not sufficient samples.

## 6. Future

This article is the first part of our researches about the intelligent recognition of navigational environment at night. In the future works, following directions will be studied further. Because the light on frames of video shoot from long distance is always small, although spatial attention mechanism is helpful to locate the position of light, when resolution of the light part is too low, it is still difficult for models to extract the features of light. So, super resolution based on GAN (Generative Adversarial Network) will be utilized as enhancement method to preprocess the low-resolution image of navigation mark's light. In addition, there are other adverse factors lowering the quality of video, light pollution from shore, and temporary block by moving vessel; they also should be treated carefully to increase the practicability of model. Furthermore, in addition to navigation marks, vessels also should be identified at night by their navigation lights, which comply with a set of complex regulations to represent different vessel's behaviors. So, it is another big challenge to recognize vessel's navigation lights and distinguish them from the lights on navigation marks.

## Figures and Tables

**Algorithm 1:**
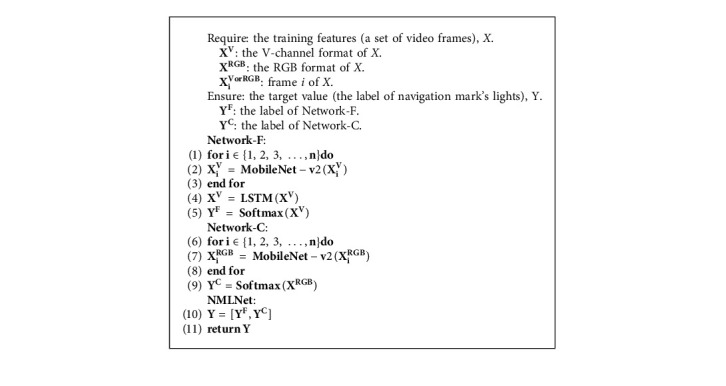
NMLNet

**Table 1:** Common types of light's phase characteristic.

Symbol	Type	Describe	Example
*F*	Fix	The light is always on.	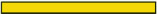
FI	Flashing	The duration of light is less than the darkness, and flashing frequency does not exceed 30 times per minute.	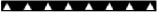
*Q*	Quick flashing	The duration of light is less than the darkness, and the flashing frequency is at least 60 times per minute.	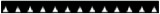
VQ	Very quick flashing	The duration of light is less than the darkness, and the flashing frequency is at least 100 times per minute.	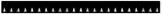
Iso	Isophase	The light consists of both a light and a dark interval with equal duration.	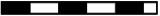
Oc	Occulting	The duration of light is more than the darkness.	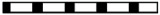
LFI	Long flashing	The light has one long flash at least 2 seconds in a period.	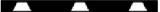
Mo(Letter)	Morse code	The light shows flashes according to Morse code.	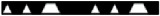

**Table 2:** Flashing labels for classification of navigation mark's lights.

No.	Label	Flashing time series	Related navigation marks
F-1	FI (1) 4s	0.80 + (3.20)	Lateral marks (port 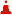 *R*, starboard 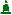 G)
F-2	FI (2) 5s	0.50 + (1.00) + 0.5 + (3.00)	Lateral marks (port 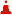 *R*, starboard 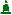 G)Marks indicating isolated dangers ( 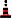 W)
F-3	FI (2 + 1) 6s	0.40 + (0.80) + 0.40 + (1.60) + 0.40 + (2.40)	Lateral marks indicating the preferred channel (port 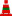 *R*, starboard 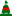 G)
F-4	Q 1s	0.50 + (0.50)	North cardinal marks ( 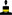 W)
F-5	Q (3) 10s	0.50 + (0.50) + 0.50 + (0.50) + 0.50 + (7.50)	East cardinal marks ( 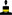 W)
F-6	Q (6) + LFI 15s	0.50 + (0.50) + 0.50 + (0.50) + 0.50 + (0.50) + 0.50 + (0.50) + 0.50 + (0.50) + 0.50 + (0.50) + 3.0 + (6.0)	South cardinal marks ( 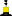 W)
F-7	Q (9) 15s	0.50 + (0.50) + 0.50 + (0.50) + 0.50 + (0.50) + 0.50 + (0.50) + 0.50 + (0.50) + 0.50 + (0.50) + 0.50 + (0.50) + 0.50 + (0.50) + 0.50 + (6.50)	West cardinal marks ( 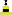 W)
F-8	LFI 8s	3.00 + (5.00)	Marks indicating safe water ( 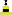 W)
F-9	Iso 4s	2.00 + (2.00)	Marks indicating safe water ( 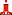 W)
F-10	Occ 4s	3.00 + (1.00)	Marks indicating safe water ( 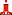 W)
F-11	Mo (*Q*) 12s	1.20 + (0.40) + 1.20 + (0.40) + 0.40 + (0.40) + 1.20 + (6.80)	Special marks for anchorage ( 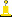 Y)
F-12	Mo (P) 12s	0.40 + (0.40) + 1.20 + (0.40) + 1.20 + (0.40) + 0.40 + (7.60)	Special marks for prohibited area ( 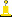 Y)

**Table 3:** Comparison of different models.

Classification	Acc	Params (M)	Latency_time (CPU)	Latency_time (GPU)
Binary relevance	**99.23** ± **0.2**	**33.66**	**1155** ± **50 ms**	**398** ± **50 ms**
Label power set	96.05 ± 0.2	35.74	1394 ± 50 ms	750 ± 50 ms
Adapted algorithm	97.08 ± 0.2	35.75	1365 ± 50 ms	759 ± 50 ms

**Table 4:** Comparison of different backbone networks.

Backbone	Acc	Params (M)	Latency_time (CPU)	Latency_time (GPU)
ResNet-50	99.40 ± 0.2	118.31	5098 ± 50 ms	1755 ± 50 ms
Mobilenet-v2	96.15 ± 0.2	33.76	1133 ± 50 ms	390 ± 50 ms
Improved Mobilenet-v2	**99.23** ± **0.2**	**33.66**	**1155** ± **50 ms**	**398** ± **50 ms**

## Data Availability

The data used to support the findings of this study are included within the article. Access to the video data set of navigation mark's lights used to support the findings of this study is restricted, because it belongs to the Changjiang Nanjing Waterway Bureau of the People's Republic of China.
